# Stercoral Colitis in a Patient With Pediatric-Onset Systemic Lupus Erythematosus: Case Analysis and Review of the Literature

**DOI:** 10.3389/fped.2021.760517

**Published:** 2021-10-27

**Authors:** Chun-Chun Gau, Li-Lun Lin, Chao-Yi Wu, Jing-Long Huang

**Affiliations:** ^1^Division of Allergy, Asthma, and Rheumatology, Department of Pediatrics, Chang Gung Memorial Hospital, College of Medicine, Chang Gung University, Taoyuan, Taiwan; ^2^Department of Pediatrics, Chang Gung Memorial Hospital, Keelung, Taiwan; ^3^Graduate Institute of Clinical Medical Science, College of Medicine, Chang Gung University, Taoyuan, Taiwan; ^4^Department of Pediatrics, New Taipei Municipal TuCheng Hospital, New Taipei City, Taiwan

**Keywords:** pediatric-onset systemic lupus erythematosus, stercoral colitis, stercoral perforation, case report, neuropsychiatric SLE (NPSLE)

## Abstract

Systemic lupus erythematosus (SLE) is an autoantibody-related disease that affects multiple organs. Stercoral colitis (SC) is a rare type of inflammatory colitis with a high mortality rate. Here, we report the first case of pediatric-onset lupus in a case complicated by stercoral colitis. We also conducted a literature review of patients with SC under 30 years old to provide useful clues for rapid diagnosis at a young age. A 28-year-old female with a history of lupus and neuropsychiatric SLE was admitted with severe abdominal pain. She was found to have stercoral colitis during surgery. Two years later, the patient underwent Hartman's operation due to ischemia of the colon. In addition, 10 patients younger than 30 years old with a diagnosis of SC were analyzed based on clinical presentation, physical examination, laboratory exam, imaging and treatment. All cases had a favorable outcome without mortality. Stercoral colitis is a rare but lethal complication, emphasizing the importance of a multidisciplinary approach. Differential diagnosis should include stercoral colitis for patients with SLE developing unexplained sharp abdominal pain.

## Introduction

Systemic lupus erythematosus (SLE) is an autoantibody-related disease that affects multiple organs. In addition, some SLE patients have gastrointestinal presentations or complications, including 6% of those with chronic constipation (1). Such involvement may be due to lupus enteritis, lupus mesenteric vasculitis, inflammatory bowel disease or adverse reactions of drugs used to treat SLE (2). Furthermore, patients with pediatric-onset lupus, accounting for 15–20% of lupus cases, have severe presentations, with 19% having gastrointestinal manifestations (3, 4).

Stercoral colitis is a rare type of inflammatory colitis with a high mortality rate ranging from 32 to 57%; it occurs mainly in elderly individuals with a history of chronic constipation, and it presents in a moribund state (5). Chronic constipation may lead to fecaloma formation in the large bowel and cause an increase in intraluminal pressure, eventually inducing bowel wall necrosis and perforation, known as stercoral perforation. Most patients are diagnosed during emergency laparotomy or post-mortem (6).

To our knowledge, this is the first reported pediatric-onset lupus case complicated by stercoral colitis. The importance of the possibility of the occurrence of this disease in patients with SLE should be highlighted, even in young adults and especially in those with early childhood-onset disease. Moreover, due to the high mortality rate of stercoral colitis, awareness is promptly required for immediate diagnosis and treatment.

## Case Presentation

A 28-year-old female was diagnosed with pediatric-onset lupus at the age of 8, with initial presentations of fever, malar rash, arthritis, positive antinuclear antibodies (ANAs), and elevated anti-dsDNA antibodies. In addition, she had episodes of neuropsychiatric SLE (NPSLE) at the ages of 19, 22, and 24, with seizures, cranial neuropathies of the facial nerve and transverse myelitis with neurogenic bladder, respectively, and treated with one dose of 0.5 g/m^2^ intravenous cyclophosphamide pulse therapy. Furthermore, she experienced a brain abscess at the age of 25 years. After the above presentations, she regained clear consciousness without cognitive dysfunction or neurologic deficits. Daily medication was maintained with 5 mg prednisolone (i.e., cumulative dose of ~144 g) and 100 mg azathioprine daily, with regular follow-up at our pediatric rheumatology clinic.

Later, she presented to our emergency department with severe abdominal pain and fever for 1 day. She had a history of chronic constipation and urinary tract infection for 2 weeks before admission, and her last bowel movement was 3 days prior. On arrival, she showed tachycardia (123 beats per min) and a body temperature of 38°C but no hypertension or hypotension (121/70 mmHg). Physical examination revealed a distended abdomen, severe tenderness in the lower abdomen, positive peritoneal signs, rebounding pain and decreased bowel sounds; rectal examination was not performed. Blood laboratory tests showed elevated inflammation markers, a C-reactive protein (CRP) level of 77.35 mg/L, a white blood cell count of 7,900/μL, and normal platelet count, serum creatinine, electrolyte and liver function test results. There were no obvious symptoms of lupus attacks, such as discoid rash, oral ulcer or arthralgia. SLE activity remained stable. C3 and C4 levels were 63.4 and 19.1 mg/dL, respectively, and her anti-dsDNA antibody level was 382.0 U/mL, lower than those over the previous 6 months. Disease activity, as quantified by the SLEDAI-2K score (7), was calculated to be 6, which indicated that a lupus flare was unlikely.

**Figure 1:**
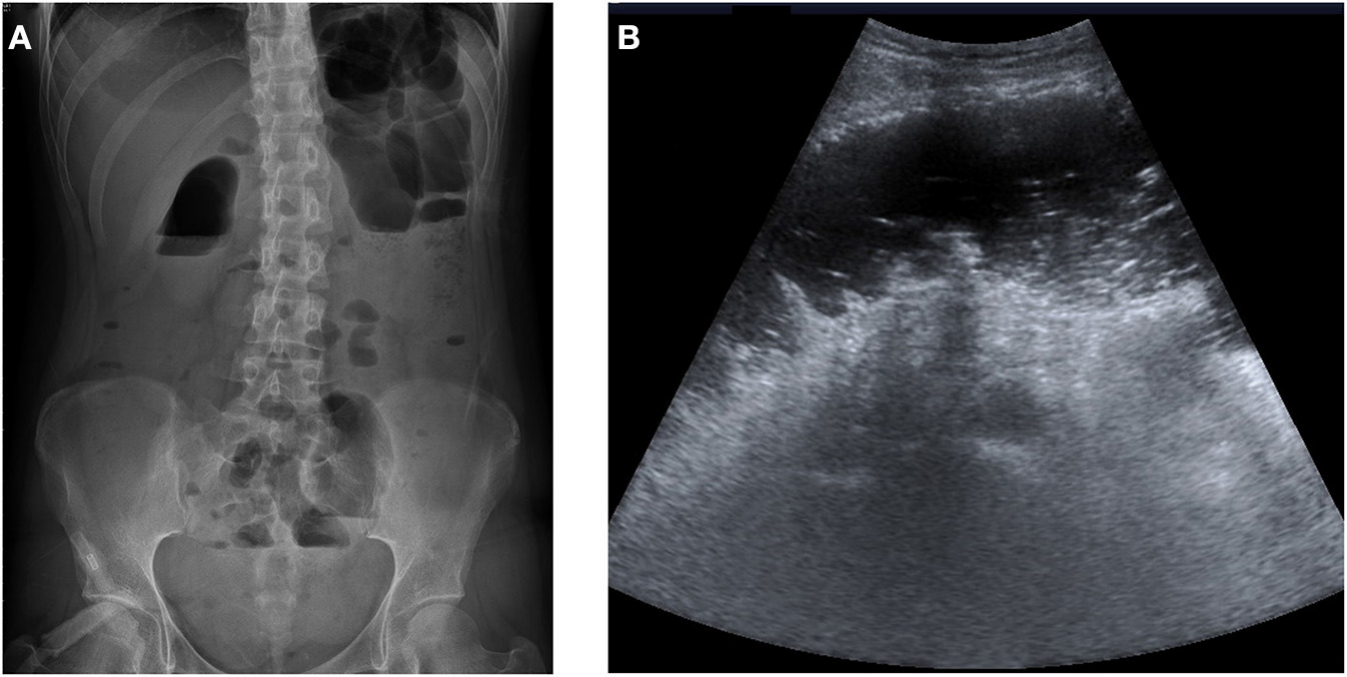
**(A)** The initial abdominal plain film showed non-specific bowel gas distribution without abnormal calcification or free air. **(B)** Ultrasonography of the abdomen revealed colon dilatation with air-fluid levels and ascites.

**Figure 2:**
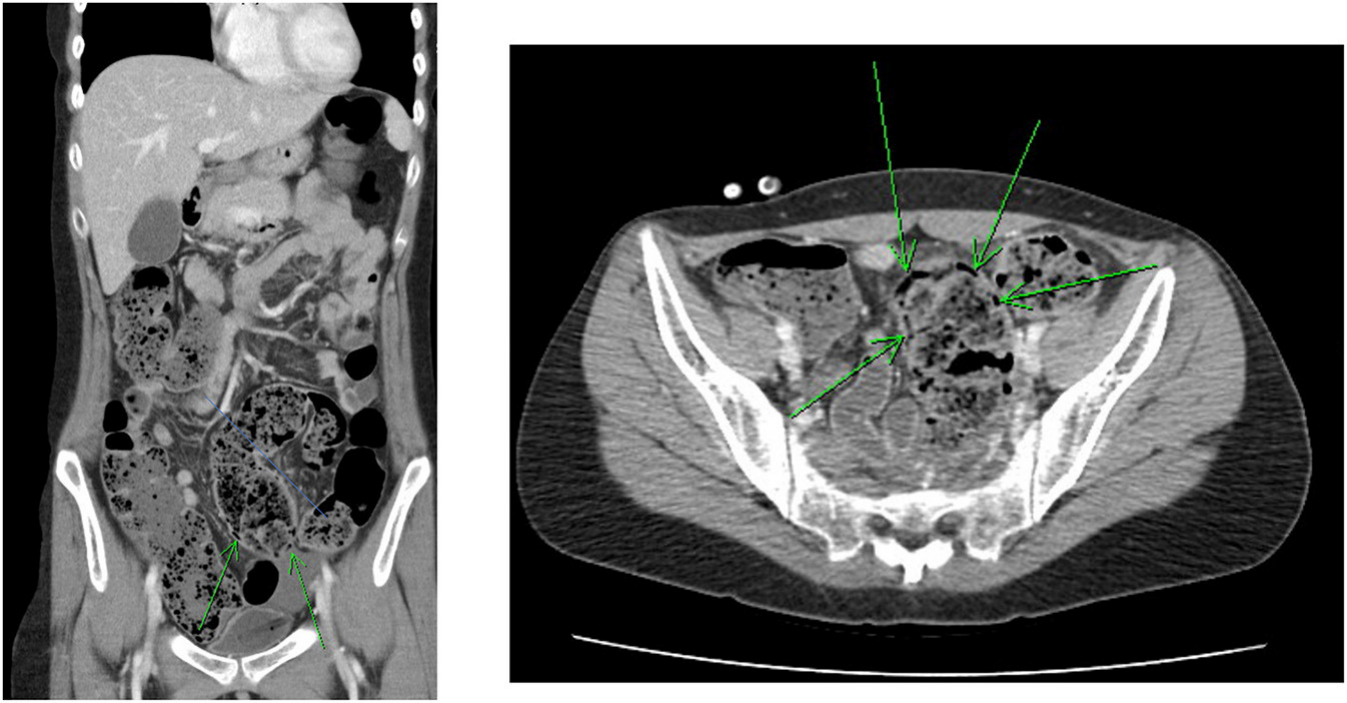
Abdominal CT revealed much fecal impaction and colon dilatation along with colon wall thickening and pneumatosis intestinalis (arrow).

**Figure 3:**
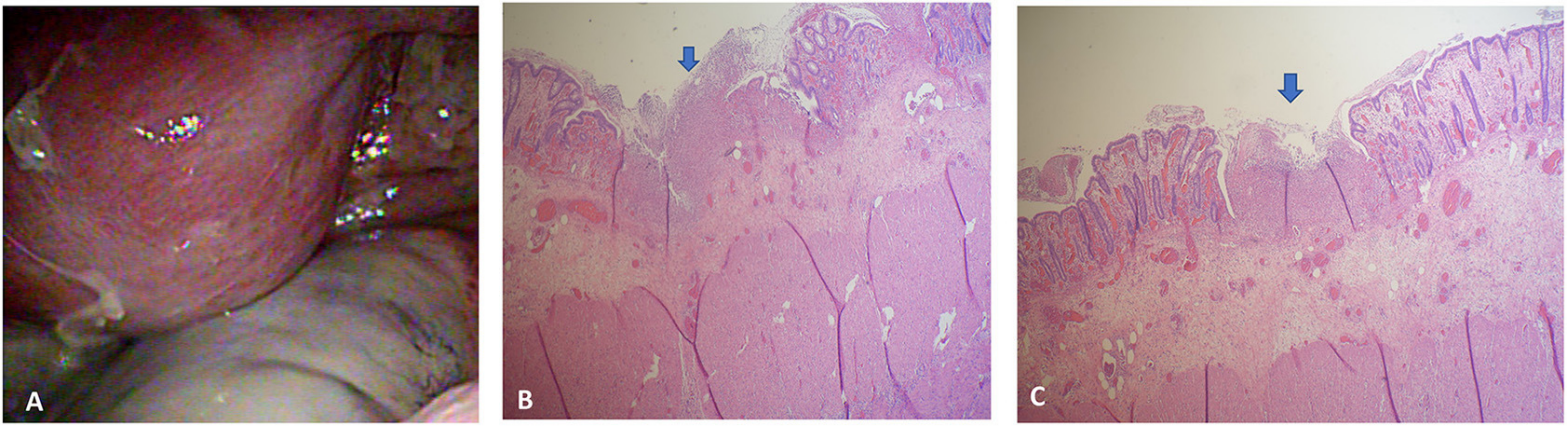
**(A)** Exploratory laparoscopy revealed poor perfusion of the upper rectum with pericolonic fibrin and turbid ascites in the pelvic cavity. **(B)** The section showed ischemic change, intensive mucosal injury, and focal transmural necrosis of the large intestine (arrow). **(C)** The specimen showed focal ischemic change and transmural necrosis of the large intestine.

The initial plain abdominal radiograph in Figure 1 shows a non-specific intestinal gas distribution without abnormal calcification or pneumoperitoneum. Ultrasound examination revealed a dilated colon, accompanied by air-fluid levels and ascites with a large amount of residual urine. A Foley catheter was inserted, but her abdominal pain did not improve. As peritonitis was suspected, she was given 1,000 g ceftriaxone and 500 mg metronidazole. However, at 6 hour after admission, the abdominal pain worsened, and repeated blood tests revealed the progression of inflammatory markers, with a CRP level of 197 mg/L. A subsequent computed tomography (CT) scan indicated a large number of fecal impactions and colon dilations, along with colon wall thickening and pneumatosis coli, as depicted in Figure 2. Laparoscopic exploration indicated poor perfusion of the upper rectum with pericolonic fibrin and turbid ascites in the pelvic cavity, but no perforation was found. Rigid sigmoidoscopy showed ischemia of the rectal mucosa. The ischemic rectum was removed, and primary anastomosis of the descending colon and rectum was performed, with a diverting loop transverse colostomy over the right quadrant of the abdomen (Figure 3). The pathology illustrated in Figure 3 showed focal ischemic changes, severe mucosal damage and focal transmural necrosis of the large intestine, which were evidence for diagnosis. All cultures were negative, and the patient was discharged at 46 days after surgery. However, 2 years later, the patient experienced abdominal pain and shock, and CT revealed inferior mesenteric artery occlusion and ischemia of the descending sigmoid colon. During the second operation, we observed ischemic colitis with gangrene changes, and the Hartmann procedure was performed. The patient recovered smoothly under our outpatient follow-up.

## Literature Review-Based Case Series

Medical records, including clinical records, physical examination results, laboratory results and imaging series, were reviewed by Lin LL and Gau CC of Taiwan's tertiary center. This study was approved by the Ethics Committee on Human Studies at Chang Gung Memorial Hospital in Taiwan, R.O.C. (IRB 201601678A3C501). Informed consent was obtained from the patient. We report this case according to CARE (for Case Reports) guidelines (8).

In a review of the literature by Lin LL and Gau CC, clinical manifestations, imaging findings, management strategies and outcomes were analyzed. We used the Medline subheading keywords “systemic lupus erythematosus (SLE),” “stercoral colitis,” and “stercoral perforation” to search for studies in PubMed published between 1965 and August 2020 in English. Relevant articles and additional references were found by checking the citations in the articles retrieved.

To date, fewer than 200 cases of stercoral colitis or stercoral perforation have been reported, and only 10 patients [3 males (9–11) and 7 females (12–17)] younger than 30 years old are reported in PubMed. The analysis of patients aged 2–28 years old is shown in Table 1. Except for the present patient, all of the young adult or adolescent patients experienced psychiatric problems or were under substance use. According to the presentations, most patients (8 of 10) complained of abdominal pain, but only 40% had fever. According to physical examinations, signs of peritonitis were detected only in six cases (cases 1, 3, 5, 8, 9, and 10). Laboratory testing showed obvious inflammatory processes in 6 patients, with 2 having metabolic acidosis and one patient both anemia and acute kidney injury. According to the indications of Kumar et al. (18), 7 cases showed diagnostic signs of stercoral perforation and CT features of fecal protrusion through the colonic wall; extraluminal air was found in half of the cases. Seven of the patients underwent surgery, and all of them were alive.

**Table 1:** Comparison of our patient with published data on stercoral colitis or perforation at young ages.

**Case**	**Patient**	**Past history**	**Presentation**	**Vital sign**	**Physical finding**	**Laboratory**	**Image**	**Operation**	**Course**
Our case (Case 1)	28 yo female	Lupus, NPSLE, brain abscess	Severe abdominal pain with fever for 3 day	BT 38°C HR 123 bpm BP 121/70 mmHg	**Abdomen** tenderness, peritoneal sign	inflammatory process	**X-ray**: non-remarkable **CT**: fecal impaction and colon dilatation with pneumatosis	Diverting loop transverse colostomy	Discharge at 46 days post-operatively Reoperation 2 years later
Hussain et al. (13) (Case 2)	28 yo female	Opioids for chronic pain	Diffuse, chronic abdominal pain, nausea, anorexia, and the complete inability to defecate for 6 weeks	Normal	**Abdomen** soft, diffusely tenderness, hypoactive bowel sound without peritoneal sign **Rectal exam**: hard mass	Unremarkable	**CT**: gastric distension and a moderate to large amount of colonic stool	No	Endoscopic decompaction and discharge
Brown et al. (12) (Case 3)	27 yo female	3-year heroin use and depression medication	1-week abdominal pain and 12-hour vomiting	HR 160 bpm RR 30/min SpO2 100% BP 90/60 mmHg	**Abdomen** distended and peritonitic	Metabolic acidosis, AKI, inflammatory process, anemia	**X-ray**: no subdiaphragmatic air **CT:** portal venous gas and free intraperitoneal fluid and gas and massive fecal distension	Hartmann's procedure perforation in the sigmoid colon	Discharged at 4 weeks post-operatively
Canders el al. (9) (Case 4)	26 yo male	Anxiety around using the restroom	Cramping abdominal pain in the lower quadrants and shortness of breath	BT 38.3°C HR 120 bpm RR 16/min SpO2 96% BP 109/81 mmHg	**Abdomen** distended and non-tender, with stool palpable in the left lower quadrant and normal bowel sounds **Rectal exam** Hard stool	Normal	**X-ray**: dilated colon with severe fecal impaction without pneumoperitoneum. **CT**: fecal impaction with bowel ischemia	No	Intravenous fluids, oral laxatives, and water enemas. Discharged on hospital day 8
Lundy and Gadacz (10) (Case 5)	25 yo male	Chronic constipation Narcotics	Severe, diffuse abdominal pain that began 3 h	Tachycardic, diaphoretic, and tachypneic	**Abdomen** rigidity with involuntary guarding.	N/A	N/A	A resection of distended sigmoid and rectum	An uneventful recovery and discharged
McHugh et al. (14) (Case 6)	17 yo female	Chronic constipation Eating disorder	24-h history of left-sided abdominal pain	HR 140 bpm BP 100/77 mmHg	**Abdomen** distended and tender across her lower abdomen without guarding or rebound **Rectal exam**: feces in the rectum	Anemia Inflammatory process	**X-ray**: severe fecal throughout the large intestine, without free air **CT**: colonic perforation, with sigmoid colon dilated to 11 cm	Total colectomy with end ileostomy Perforation at the sigmoid colon without fecaloma	Repeated laparotomy
Proulx and Glass (15) (Case 7)	9 yo female	Chronic constipation	Nausea, vomiting, and diarrhea for several hours	BT 97.2°F (36.2°C) HR 154 bpm RR 22 bpm SpO_2_ 95% BP 70/50 mm Hg SpO_2_ 95%	**Abdomen** palpable suprapubic mass, mild generalized tenderness without peritoneal sign **Rectal exam** normal tone with a large stool mass	Leukocytosis Metabolic acidosis	**CT** large stool burden in the rectum, comprising a mass of ~7 cm	No	Fluid supplement, manual decompaction, antibiotics, anorectal irrigation every 6 h. Discharged on hospital day 8
Park et al. (11) (Case 8)	6 yo male	Ehlers-Danlos Syndrome	Abdominal pain for 4 days without nausea, vomiting, or fever	Tachycardic Normotensive	**Abdomen** tenderness to percussion, pain with movement, and voluntary guarding	Normal	**X-ray**: unremarkable **CT**: free fluid with peripheral edema below the kidneys	Loop colostomy perforation on the lateral mesenteric border of descending colon	Discharged on day 11 post-operatively However, reoperation at 14 months later
Huang et al. (17) (Case 9)	4 yo female	Chronic constipation	Sudden and severe abdominal pain	Fever	**Abdomen** diffuse peritonitis	Leukocytosis	**X-ray**: Subdiaphragmatic air	Segmental colectomy Perforation at antimesocolic site over mid-sigmoid Colon	No complication
Al Omran et al. (16) (Case 10)	2 yo female	Overdose of Ibuprofen	a 3-day history of cough, fever and general aches	BT 39.1°C HR 155 bpm RR 50/min BP 90/34 mmHg	**Abdomen**: distended, with guarding and rebound tenderness	Leukocytosis	**Coronal anteroposterior CT:** free air and abundant fecal in the pelvis **Contrast-enhanced axial CT:** portal and retroperitoneal air	Double-barrel colostomy Perforation on the antimesenteric side of the sigmoid colon	Discharged a week after surgery

*NPSLE, neuropsychiatric systemic lupus erythematosus; yo, year-old; HR, heart rate; RR, respiratory rate; SpO_2_, saturation oxygen; BP, blood pressure; AKI, acute kidney injury; N/A, not applicable; BT, body temperature*.

## Discussion

Stercoral perforation is a rare but fatal complication of constipation and fecal impaction. Before our patient, fewer than 200 cases have been reported since the first report in 1894 (19). Maurer et al. asserted that 1.2% of all emergency colorectal procedures and 3.2% of all colonic perforations involve stercoral perforation (6). In a retrospective study, stercoral perforation occurred in 81% of patients, especially with relation to a long-term history of chronic constipation with a median age of 62 (19). In general, patients taking medication such as non-steroidal anti-inflammatory drugs (NSAIDs) have a higher risk of stercoral perforation (20, 21) due to the propensity of these drugs to travel slowly and transiently through the bowel.

By exploring the published English literature (PubMed search from 1965 through August 2020), we found only two cases of stercoral colitis in patients with SLE (22, 23). In one case report, a 45-year-old woman with SLE presented with epigastric pain for 12 hours, and the author believed that the cause of stercoral perforation was related to NSAIDs (22). Another case report involved a 44-year-old SLE woman presenting with stercoral perforation due to long-term steroid use (23). Our patient is the first pediatric-onset (onset age at the 8) lupus case complicated by stercoral colitis without a disease flare or NSAID medication use. Although there is a reports that ischemia colitis is associated with antiphospholipid syndrome (24), but reviewing her 20-year history of lupus, no antiphospholipid antibodies were found. In addition, it is reported that some anticonvulsants can induce stercoral colitis (25), but only one short-term benzodiazepines was used 9 year ago to control a seizure. Importantly, we mainly considered the four neurological events (seizure, cranial neuropathy, transverse myelitis, and brain abscess) lead to impaired nerve innervation and cause neurogenic bladder and chronic constipation, which resulted in this catastrophic stercoral colitis. The sigmoid colon and rectum, especially the rectosigmoid junction, are the most vulnerable parts of the colon for stercoral colitis development. An insufficient blood supply at the rectosigmoid junction is specifically defined as Sudeck's point with insufficient or absent anastomosis between the superior rectal artery and inferior mesenteric artery branch at the watershed area (17, 26). Although her lupus condition was stable, bowel inflammation may also have contributed to poor tissue recovery in this insufficient blood supply area and the need for a second operation. In terms of the multiple manifestations of lupus, a correct diagnosis is difficult in this population.

Various causes of abdominal pain in lupus may indicate misdiagnosis, such as lupus enteritis, primary peritonitis and chronic lupus peritonitis (27, 28). Lupus enteritis, including mesenteric arteritis or vasculitis, gastrointestinal vasculitis and intestinal vasculitis, is a rare but life-threatening complication of SLE. Furthermore, postprandial abdominal pain can be insidious due to chronic mesenteric ischemia (27). Primary peritonitis secondary to SLE can develop rapidly, but the simultaneous use of immunosuppressive agents might mask the symptoms. In view of the aforementioned challenges of misleading diagnosis, stercoral colitis is easily overlooked, but prompt treatment is required due to its rapid progression.

According to Maurer et al.'s definition (6), there are three diagnostic criteria: (1) a round or ovoid colonic perforation more than 1 cm in diameter on the antimesenteric side; (2) fecalomas observed in the colon, protruding through the perforation site or lying within the abdominal cavity; and (3) pressure necrosis or ulceration with chronic inflammatory reactions around the perforation site. In addition, it has been reported that the mortality rate is 32–60%, regardless of age (5). In our analysis, all patients met the diagnostic criteria, were still alive and had been discharged from the hospital; thus, a young age may result in favorable outcomes.

Early diagnosis of stercoral colitis remains a dilemma. According to a previous review, not all cases involve fever, peritoneal signs, elevation of inflammatory markers, metabolic acidosis, or electrolyte imbalance (16, 29). In addition, urinary retention, incontinence or frequency may be early signs of fecal obstruction (30). A tubular mass may be palpable in the lower left quadrant because the fecal-filled rectosigmoid and rectal exam can reveal feces located at the sigmoid colon and obstruction (31, 32). One of the patients in our review was at the age of two and unable to express abdominal discomfort; therefore, diagnosis may be difficult to confirm. Our lupus patient had urinary retention, which should be one of the early signs of chronic constipation. Our review is also in line with previous research showing that peritoneal signs and inflammation laboratory data were observed in only 60% of cases.

Although previous experts have mentioned that pain during an upright abdominal X-ray examination can provide clues about stercoral colitis or perforation, including colonic swelling at the impaction site, calcified fecaloma, or free air, fecal matter may obscure these findings (6, 33). The pivotal diagnostic role of CT radiologic investigation includes a large fecaloma with distention >6 cm of the affected colon, wall thickening >3 mm of the affected colon, pericolonic fat stranding, mucosal discontinuity, free fluid, pericolonic abscess and extraluminal gas bubbles (18, 33–35). However, in our review of patients at a young age, plain abdominal X-ray did not reveal remarkable findings for most patients, with only half displaying perforation on CT images.

According to Wu et al.'s research, 52% of patients can be treated non-operatively through an intestinal regimen (36). However, if peritonitis occurs, emergency laparotomy should be performed to rule out situations that may be related to stercoral perforation. Stercoral colitis can be diagnosed through pathology and intraoperatively (6). The usual surgery is Hartmann's procedure, which removes possible lesions (37). Intraoperative colonoscopy should be applied to determine the presence of additional stercoral ulcers, and a specimen of the altered or dilated colon should be taken to prevent recurrence of stercoral perforation (17). Additionally, pathology can reveal mucosal hemorrhage, submucosal congestion, and sharp demarcation without undermining the ulcer margins and transmural necrosis on the perforated side (29, 36). In our young age case analysis, 30% of patients received conservative treatment with good prognosis but two cases need re-operation. One was our patient and the other was a patient with Ehlers-Danlos syndrome, a connective tissue disease, and both underlying diseases may cause poor wound healing.

## Conclusion

It is important to identify SLE patients with severe abdominal pain who may be at risk of stercoral colitis or perforation. Stercoral colitis is a rare yet lethal complication that can occur in patients as young as 28 years old, and differential diagnosis should highlight the importance of a multidisciplinary approach. In addition to appendicitis, peritonitis, or vasculitis, differential diagnosis should include stercoral colitis for patients with SLE who develop unexplained sharp abdominal pain.

## Data Availability Statement

The original contributions presented in the study are included in the article/supplementary material, further inquiries can be directed to the corresponding author.

## Ethics Statement

The studies involving human participants were reviewed and approved by Ethics Committee on Human Studies at Chang Gung Memorial Hospital in Taiwan, R.O.C. (IRB 201800989A3). Written informed consent to participate in this study was provided by the participants' legal guardian/next of kin.

## Author Contributions

C-CG and L-LL carried out the case analysis, participated in the sequence alignment, and drafted the manuscript. C-YW and J-LH participated in the design of the study and performed the statistical analysis. J-LH conceived of the study and participated in its design. All authors contributed to the article and approved the submitted version.

## Funding

This work was supported by the Research Fund of Chang Gung Memorial Hospital CMRPG3H1201-2, CMRPVVH0023, the National Science Council NMRPVVK6021-3 and the Ministry of Science and Technology (MOST; 109-2314-B-182A-105-MY3).

## Conflict of Interest

The authors declare that the research was conducted in the absence of any commercial or financial relationships that could be construed as a potential conflict of interest.

## Publisher's Note

All claims expressed in this article are solely those of the authors and do not necessarily represent those of their affiliated organizations, or those of the publisher, the editors and the reviewers. Any product that may be evaluated in this article, or claim that may be made by its manufacturer, is not guaranteed or endorsed by the publisher.

## Abbreviations

ANAs: antinuclear antibodies
CRP: C-reactive protein
CT: computed tomography
NSAIDs: non-steroidal anti-inflammatory drugs
NPSLE: neuropsychiatric SLE
SC: stercoral colitis
SLE: systemic lupus erythematosus
WBC: white blood cell.
